# The sudden switch to online communication training after 10 years in the classroom – comparing the evaluation results of a course on doctor-patient communication

**DOI:** 10.3205/zma001543

**Published:** 2022-04-14

**Authors:** Miriam Schwär, Judith Ullmann-Moskovits, Maria Farquharson, Monika Sennekamp

**Affiliations:** 1 Goethe University Frankfurt am Main, Institute of General Practice, Frankfurt am Main, Germany

**Keywords:** blended learning, health history intervie, communication, communication with patients

## Abstract

**Objective:**

The aim of the study was to find out whether it is possible to successfully convert a communication course for around 400 students to a blended-learning format (asynchronous theoretical course/synchronous digital practical course). The main focus thereby was on assessing subjective learning progress and the extent to which the importance of communication and doctor-patient communication can be conveyed online. The study is based on the results of an evaluation of the opinions of both the students and the lecturers that participated in the course.

**Methods:**

The students, who were in their fourth preclinical semester in 2020, were asked to fill in a self-assessment sheet at the beginning of the course, and following its completion. The feedback provided by the lecturers was also assessed. In order to compare the results and identify possible discrepancies, the corresponding self-assessment and evaluation results for the past 10 years (stemming from traditional classroom courses) were also taken into account.

**Results:**

Participants in the online courses reported distinct subjective learning progress, and greater progress than was reported for traditional courses in previous years. The suitability of the online format was viewed critically by both students and lecturers, while the course atmosphere was seen positively. The relevance of doctor-patient communication was assessed particularly highly in the online format.

**Conclusion:**

Based on the results of the evaluation, the experience gained from the blended-learning format will be included into future iterations of the communication course at Goethe University Frankfurt. The results have shown that doctor-patient communication can be learned well online. This format can therefore be recommended for new learning concepts in the future.

## Zusammenfassung

**Zielsetzung:**

Ziel der Studie ist die Frage, ob es möglich ist, einen Kommunikationskurs für ca. 400 Studierende erfolgreich in ein Blended Learning-Format (asynchrone Theorievermittlung/digitaler synchroner Praxisteil) umzustellen. Hauptfokus war dabei die Beurteilung des subjektiven Lernzuwachses und die Frage, inwiefern die Bedeutung des Themas Kommunikation und Gesprächsführung online abgebildet werden kann. Die Betrachtung erfolgt auf Basis von Evaluationsergebnissen der Studierenden und Dozierenden dieses Kurses.

**Methodik:**

Die Studierenden des vierten vorklinischen Semesters aus dem Jahr 2020 wurden durch einen Selbsteinschätzungsbogen zu Beginn sowie durch eine Evaluation nach Abschluss des Kurses befragt. Zusätzlich wurden die Rückmeldungen der Dozierenden zum Kurs ausgewertet. Um die Ergebnisse vergleichen und mögliche Unterschiede feststellen zu können, wurden die entsprechenden Selbsteinschätzungs- und Evaluationsergebnisse der vorangegangenen zehn Jahre (in Form von Präsenzkursen) herangezogen.

**Ergebnisse:**

Im Onlineformat wurde ein deutlicher subjektiver Lernzuwachs angegeben, der höher ausfiel als in den Vorjahren in Präsenz. Die Eignung des Onlineformats wurde von Studierenden sowie Dozierenden eher kritisch bewertet, während die Kursatmosphäre positiv gesehen wurde. Die Relevanz ärztlicher Gesprächsführung wurde im Onlineformat insgesamt besonders gut bewertet.

**Schlussfolgerung:**

Aufgrund der Evaluationsergebnisse werden die Erfahrungen mit dem Blended Learning-Format auch in Zukunft in das Kommunikations-Curriculum des Medizinstudiums der Goethe-Universität Frankfurt einfließen. Die Ergebnisse haben gezeigt, dass das Erlernen ärztlicher Gesprächsführung online gut möglich ist. Daher kann dieses Format für zukünftige neue Konzepte empfohlen werden.

## 1. Introduction

Opportunities offered by the digitalization of teaching in medical school have been discussed in Germany for many years [1], [2], [3]. Blended learning – as well as flipped classroom events – have been used to teach adults for several years and would appear to be suitable for use in the development of online and partially online course units [4], [5]. Previous to the summer semester of 2020, however, only a few published projects on doctor-patient communication had used digital teaching formats and content [6], [7], [8], not least because medicine is largely involved with human interaction, and communication generally takes place face-to-face. In a systematic review published in 2019, Kyaw et al. conclude with respect to possibilities to use blended learning in communication courses for medical students that “blended digital education seems to be at least as effective as and potentially more effective than traditional learning for communication skills and knowledge“ [9]. Nevertheless, such teaching events, especially in courses aimed at teaching competence in communication, have not yet established themselves in the medical curriculum. 

However, the COVID-19 pandemic meant that nearly all universities in Germany had to shift to the use of digital formats from the summer semester 2020 onwards. This change also affected the course on health history taking and doctor-patient communication that was part of the “Introduction to Clinical Medicine” conducted in the fourth preclinical semester. 

## 2. Project description

The first course in the curriculum on doctor-patient communication is provided to almost 400 medical students and traditionally held in the form of seven two-hour in-class modules consisting of theoretical and practical teaching units. Comprehensive embedded feedback was used to evaluate the course [10]. On this basis, the 2020 course was converted to a blended learning format that divided the course into an asynchronous theoretical part (made available on the OLAT learning platform) and a synchronous online practical module (taught by clinical lecturers) for practicing health history interviewing. The interviews were practiced through role play in the online practical modules held via video conferencing systems with (simulated) patients and subsequent structured feedback. Key objectives of the course in 2020 and before were that students should learn the theoretical basics of communication and history taking, and then independently take a patient’s health history, which was then subjected to systematic analysis, constructive feedback as well as reflection, and in addition to the theoretical part presented the criteria for passing the course. An interdisciplinary team developed the new blended learning teaching format. Theoretical content was taught by means of asynchronous self-study that included knowledge tests and free-text exercises, video sequences (including examples of history taking), and reflective tasks aimed at ensuring the format was as lively as possible and that students could participate when it suited them. The separation of theory and practice meant the synchronous units could focus on exercises and the simulation of history taking. Furthermore, activating methods were used to create a pleasant learning atmosphere in the online groups. A new, structured process for the discussion and feedback elements was also developed for the online setting to ensure that the time spent as a group could be used as efficiently as possible [11]. 

In the literature, “emergency remote teaching“ (ERT) has been distinguished from “online teaching“ since the beginning of the COVID-19 pandemic. ERT is regarded as temporary and a kind of emergency conversion, while online teaching is regarded as an independent teaching model that involves a great deal of work and for which preparation can take up to one year. ERT was typically employed in the summer semester of 2020, while pure online teaching was rarely used [12]. Our blended learning format combines the strengths of traditional classroom learning with the possibilities offered by online teaching. As the core element of the course, health history interviews had to remain synchronous but in a form that was suitable for use in the pandemic. Rather than simply making course material available online, as foreseen by ERT, we therefore decided to develop an independent online teaching concept. 

To check the suitability of the newly developed blended learning format for teaching, the course was evaluated, as it was in previous years. Both the students (before and after the course) and the lecturers (after the course) were asked for their opinions. Learning progress and whether learning objectives had been reached were not assessed by means of an objective examination at the end of the course. Since the initial implementation of the online course, students' subjective learning progress has thus been assessed using an item in a survey. At the same time, associated factors, such as a subjective assessment of one’s own ability to take a health history independently, a subjective evaluation of the relevance of content on discussions with patients and communication as part of medical studies, the importance of theoretical background information on this subject, the suitability of the online format for learning these skills, as well as the atmosphere in the course as a basic condition for an appropriate learning environment, were also assessed [13]. The aim of this study is thus to answer the following question: Is our blended learning format for a course in doctor-patient communication suitable for enabling students to subjectively feel they have made or can make progress towards achieving their learning objectives. We also considered further factors to describe how the course was converted to an online format in order to investigate whether and to what extent the importance of communication and doctor-patient communication can be illustrated online. 

In this paper, we present the results for the year 2020 and compare them with the evaluation results for the traditional in-class format used during the previous ten years (2010-2019). 

## 3. Methods

### 3.1. Course methods

The course was conducted by medical lecturers from various clinical and theoretical departments for around 400 students, divided into groups of about 14 participants. The covered topics included communication theory, questioning techniques and active listening. All students independently conducted at least one health history interview with a (simulated) patient. 

The synchronous part of the course was conducted using the video conferencing platforms Zoom and Webex. The participating patients were informed in advance and trained in the use of the platforms where necessary. The simulated patients received training in their roles in advance. Special features of the video format were explained to them and help was provided in using the technology. 

The self-study unit of the blended learning module consisted of scripts, films, tasks and self-reflection questions. Participation was checked based on special tasks and verified by a student assistant and a researcher. The students required an average of around seven hours to prepare the content. 

#### 3.2. Evaluation methods

The data presented here are based on the three self-developed questionnaires that were used between 2010-2020: 

At the beginning of each course, all students were issued with a self-assessment sheet. Before the online semester was introduced, the sheets were provided in paper form at one of the seminars. In 2020, however, they were provided digitally in the form of a survey on the OLAT online learning platform. 

At the end of the semester, a questionnaire for the final evaluation was provided for a second assessment. From 2010-2019, this was provided in paper form when the course credit certificates were issued several weeks after the course was over. In 2020, they were provided as a LimeSurvey link in an email. 

As part of the final module and after the course had been completed, the lecturers were asked to assess the course and its relevance to overall doctor-patient communication. When the course was taught in the classroom, this was carried out on paper, whereas in 2020 the lecturers were sent a LimeSurvey link in an email. 

The evaluation data were combined and an overall evaluation of the module was conducted. The students’ evaluation sheets consisted of 22 questions (17 closed, 5 open), while the lecturers’ sheets consisted of 14 questions (8 closed, 6 open). For the online format, 7 questions were added to the students' questionnaires and 6 to the lecturers'.

The presented data were analyzed descriptively using SPSS (IBM SPSS 25.0). All items were rated on a six-point Likert scale. The possible responses ranged from 1 (very good) to 6 (very bad). 

## 4. Results

### 4.1. Self-assessment sheets

From 2010-1019, there were large differences in the response rate, which varied from 35.8% - 93.9%. In 2020, it was 84.2%. The number of missing values on the individual items varied between 0 and 8. To ensure clarity, these are not provided in detail in table 1 (Tab. 1).

#### 4. 2. Student evaluation

From 2010-1019, the response rate among students (S) varied from 25.1% - 91.6%, and the number of missing values varied between 0 and 20. In 2020, the response rate was 45.1%, while the number of missing values was 43 and noticeably higher than in previous years. 

#### 4. 3. Lecturer evaluation

From 2010-2019, the response rate among lecturers (L) varied from 15.8% - 66.7%, but was 67.6% in 2020. The number of missing values was either 0 or 1 (see table 2 (Tab. 2)).

#### 4.4. Presentation of results

**Figure 1:**
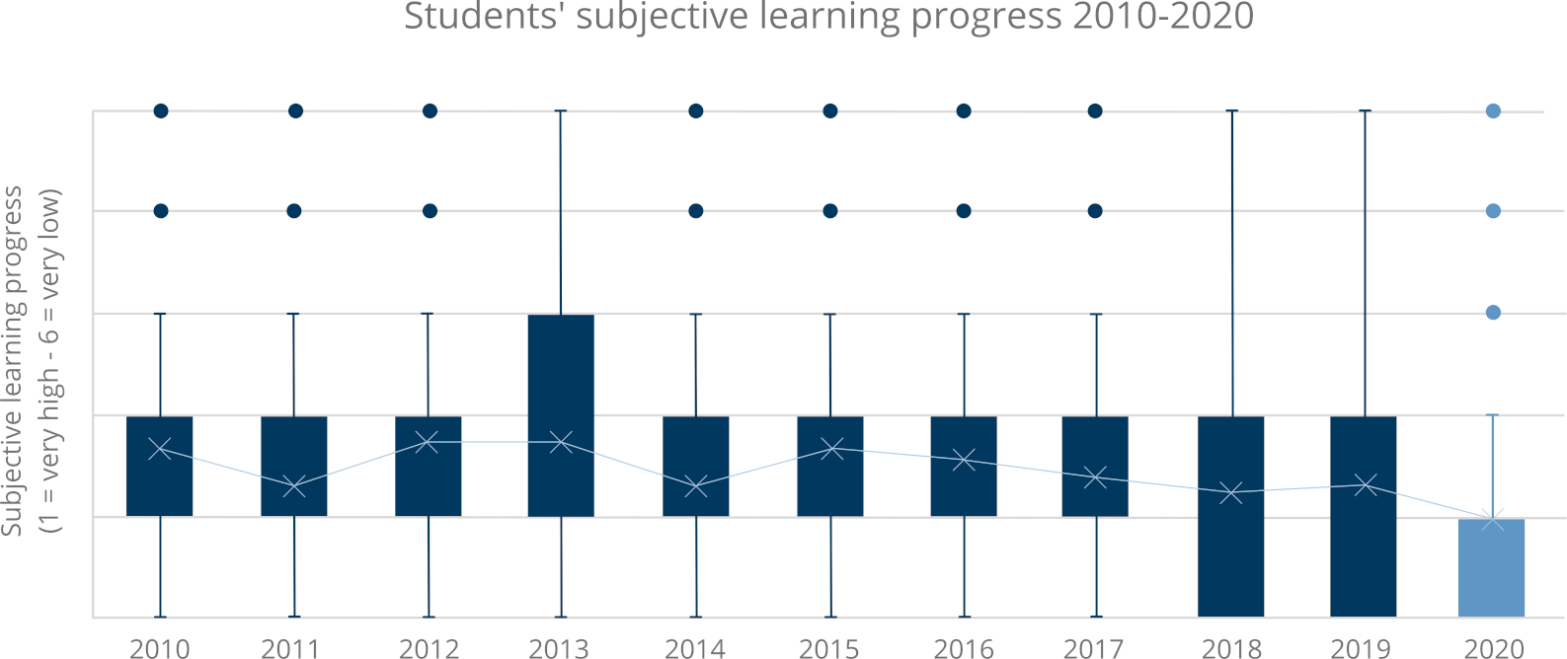
Comparison of students‘ subjective learning progress in the years 2010-2020 on a six-point Likert scale from 1=very high to 6=very low.

In the final evaluation, the subjective estimate of learning progress among students (see table 1 (Tab. 1) and figure 1 (Fig. 1)) was an average of 1.96 in 2020, or higher than in any of the previous years. 

When the students were asked whether they felt capable of “conducting a health history interview” before and after the course, the value was similar to the years 2010-1019. 

The students’ general assessment of the relevance of communication and doctor-patient communication was slightly higher before the course began in 2020 than in previous years (mean=1.23 in 2020 vs. 1.32 in 2010-2019). After the course, this value was also slightly higher than in the years when the course was held in the classroom (mean=1.25 in 2020 vs. 1.47 in 2010-2019). Among the lecturers, the overall importance of the course in medical education was also considered to be very high (mean=1.05 in 2020 vs. 1.35 in 2010-2019). 

As an important criterion for a successful learning environment and essential to role play, the assessment of the atmosphere in the course was carried out for the first time in 2019. Both in 2019, when teaching was in-class, and in 2020, when it was online, the atmosphere in the course groups appears to have been very good (mean=1.40 in 2019 vs. 1.48 in 2020).

Views on the importance of theory teaching and the opinion that it provides the foundation for medical practice play a substantial role in subjective estimates of learning progress and the question of the value of communication overall. Before the course, the assessment was a little worse than in previous years (mean=2.21 in 2020 vs. 2.15 in 2010-2019). However, in the final evaluation of the blended learning format in 2020, students’ views of the relevance of theory were slightly more positive than in previous years (mean=1.93 in 2020 vs. 2.18 in 2010-2019), as were those of the lecturers (mean=1.59 in 2020 vs. 1.94 in 2010-2019). Furthermore, both final assessments were more positive than in any of the previous 10 years.

As part of the final evaluation, both the students and the lecturers assessed the suitability of the online format for learning how to conduct health history interviews (mean S=3.06/mean D=2.87). Although the lecturers considered the structure of the course to be good overall (mean=2.35 in 2020), their assessment was less positive than in the previous 10 years (mean=1.54 in 2010-2019).

## 5. Discussion

The evaluation results in our comparative study of 10 years surveying traditional communication courses held in the classroom and a survey of a blended learning format conducted online provided a distinguished answer to the question whether the online setting is more suited to enabling subjective learning progress and to explaining the importance of the topics of overall communication and specifically doctor-patient interviews. Unlike Fischbeck et al., we do not consider the question whether online courses could or should replace courses held in the classroom in our study [8].

### 5.1. Discussion of results

It is noteworthy that mean subjective learning progress was considered to be higher than in any of the previous 10 years. In the final evaluation, further assessments, such as those of the subjective relevance of doctor-patient interviews and overall communication for the students' future careers, were viewed more positively by both students and lecturers than in any of the previous years. Perhaps contrary to expectations, the results show that the importance of communication in medical studies did not disappear due to the digital setting, but was actually considered to be higher than ever before. 

It is particularly interesting that students assessed courses in underlying communication theory more positively in the final 2020 evaluation than in previous years. However, it is worth considering that the blended learning format provided a standardized learning approach that was not dependent on individual lecturers, but that in comparison to traditional in-class learning, self-study demanded a different, more personal and intensive examination of theoretical learning content. The positive assessment of the online communication course overall is consistent with insights reported in recent publications by Kunisch et al. [14] und Mohos et al. [15], whereby the latter expressed the view that online courses might complement in-class courses. 

The decision to provide synchronous video interviews was supported by the fact that students’ confidence in their ability to conduct health history interviews remained high. It also showed that it was the subjective view of the students that the most important fundamentals could be learned in an online setting, even if participants' overall assessment of the online setting was rather critical. In this respect, the comparatively positive assessment of the course atmosphere undoubtedly played a role. Nevertheless, it is important to be aware of the limitation that the differing formats meant it was not possible to directly compare them, and the data could only present the subjective views of students. However, it is worthy of note that both the synchronous online course unit as well as the in-class courses showed similarly positive results with respect to the atmosphere. 

#### 5.2. Limitations

Overall, the comparison between one year using a blended learning format with ten years of traditional in-class courses must be viewed critically, not least because of the different sample sizes of 368 students and 34 lecturers in contrast to 3,694 students and 472 lecturers. 

As results of the blended learning format evaluation only exist for one year, we can only report preliminary tendencies. It is not possible to transfer our results to other similar formats, or to make sweeping generalizations. For this purpose, further comparative evaluation data for future student cohorts would be necessary. 

Further diverse factors that we could not measure directly may also have influenced our results. For example, we only investigated the first year of an online semester that occurred in response to the pandemic and was impacted by an array of underlying conditions that will certainly have influenced the evaluations. For example, in their free-text responses to questions asked in the survey, many students expressed considerable gratitude for the way the course was implemented in comparison to other formats employed at the time. This may have distorted our results in a positive sense. 

In this respect it is also important to consider the special features of “Media Comparison Studies” (MCS) and the possible pitfalls associated with them. In MCS, specific features of the particular medium under review are often ignored when, for example, online and traditional in-class formats are directly compared with one another [12]. The various variables cannot be broken down into distinguishable elements, and this is a limitation described in MCS. We were therefore unable to precisely compare the two formats. 

Offergeld et al. [16] describe how the pandemic led more to the emergence of “emergency distance learning” than the actual development and provision of online learning. Contrary to this view, we believe our approach, which was proposed by an interdisciplinary team, enabled us to develop a blended learning format that corresponded to a traditional classroom-based communication course from both a didactical and a technical perspective. Based on the students’ assessments of their subjective learning progress and the relevance of communication to medical studies, we consider our online course to be well suited to achieving its learning objectives. 

In order to answer our original question of whether it is possible to successfully convert a communication course to a blended learning format, it is preferable that the communication skills and learning progress of students are objectively examined after both online and in-class teaching units. 

## 6. Conclusions

Despite several limitations, it can be seen that the comprehensive research design, which included continuously large sample sizes, a long survey period and the inclusion of different points of view (students and lecturers), enabled us to present extensive data on an online communication course provided as part of medical studies. 

Contrary to the view that the blended learning format would only be employed temporarily for as long as the pandemic lasted, which we still held in 2020, a review of the data raises the question whether parts of the new implementation of the course should be retained. Based on the results of the evaluation presented here, we continued to provide the course in a virtually unchanged format in 2021. When it is again possible to provide in-class teaching, it is conceivable that we will provide a blended learning format that includes both asynchronous, digital training in theoretical fundamentals, while health history interviews will be practiced face-to-face. The results of this survey and upcoming assessments will be taken into account when designing a course in communication at Goethe University Frankfurt, whereby special consideration will be given to the restructuring of medical studies foreseen by the new licensing regulations for doctors and the increased importance they attach to communication skills. In this respect, the results of our study offer substantial scope for the use of innovative formats. 

## Note

The authors Miriam Schwär and Judith Ullmann-Moskovits share the first authorship.

## Competing interests

The authors declare that they have no competing interests. 

## Figures and Tables

**Table 1:**
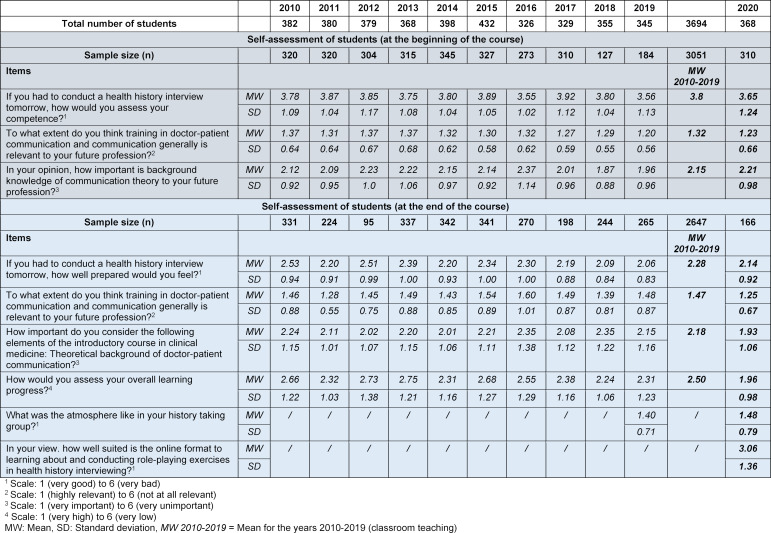
Self-assessment and final evaluation of students in 2010-2020

**Table 2:**
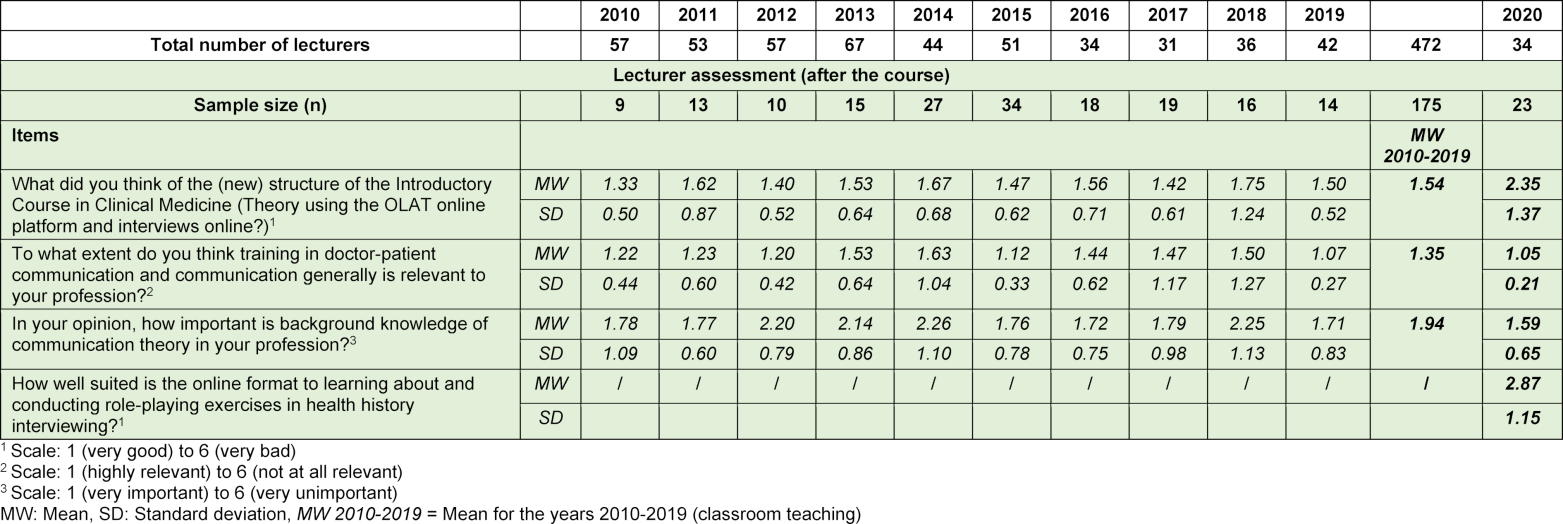
Final assessment of lecturers in 2010-2020
